# Herba Epimedium (Horny Goat Weed) Toxicity With Severe Muscle Spasms and Elevated Creatine Kinase and Creatinine: A Case Report

**DOI:** 10.7759/cureus.84608

**Published:** 2025-05-22

**Authors:** Tucker Ledo, Dhara Patel, Wayne Tamaska, James Espinosa, Alan Lucerna

**Affiliations:** 1 Emergency Medicine, Jefferson Health, Stratford, USA

**Keywords:** elevated creatine kinase from horny goat weed, elevated creatinine from horny goat weed, herba epimedium and severe muscle spasms, herba epimedium toxicity, horny goat weed and severe muscle spasms, horny goat weed toxicity, severe muscle spasms

## Abstract

Here, we present the case of a 33-year-old man who presented to the emergency department (ED) with a complaint of severe muscle spasms of a 10-hour duration. He noted that he took a supplement which he purchased over the internet called *Epimedium*, also known as horny goat weed, for the purpose of improving his mood and sense of energy and reducing his sense of anxiety. He started this supplement one month prior to ED presentation, coincident with the first episodes of muscle cramps. In the ED, he had elevations of his creatine kinase (CK) and creatinine and required hospital admission. His creatinine and CK elevations and muscle spasms improved during his hospital admission. He was discharged from the hospital with instructions to stop taking *Epimedium* supplements. *Epimedium* contains a wide variety of chemicals that vary somewhat by individual species. To our knowledge, this is the first specific case report of severe muscle spasms with associated elevations of CK and creatinine caused by *Epimedium* use. This case reinforces the importance of asking a patient about all medications, including over-the-internet, over-the-counter, and herbal medications.

## Introduction

*Epimedium* species are flowering plants of the family Berberidaceae [1]. Over 50 species of *Epimedium* have been identified [2]. The majority of the species are endemic to China, but the plant is also found in Asia and the Mediterranean region. Species of *Epimedium* are hardy perennials with four-parted flowers in the spring. The appearance of the flower has led to alternate names, such as the fairy-wing plant and the bishop's hat plant. The traditional Chinese name is yingyanghuo (ying yang huo). Extracts of plant have been used for centuries in China and in other countries for a variety of uses, including as an aphrodisiac, leading to common reference to *Epimedium* species as "horny goat weed" [1].

The plant contains a wide variety of chemicals that vary somewhat by individual species of *Epimedium*. Over 200 chemical constituents have been identified in various species of *Epimedium*, including flavonoids, lignans, ionones, phenol glycosides, and sesquiterpenes [1]. *Epimedium* species have been studied in reference to possible anti-inflammatory, immunomodulatory, antiviral, and hepatoprotective properties [2]. 

## Case presentation

A 33-year-old man presented to the emergency department (ED) with a complaint of muscle spasms of a 10-hour duration. He described the muscle spasms as extremely painful and occurring throughout his body. He was initially evaluated at an urgent care center and was prescribed cyclobenzaprine, which he took without relief. He came to the ED because his symptoms persisted. He had a past medical history of post-traumatic stress disorder (PTSD), anxiety, and vitamin D deficiency. He denied any history of smoking, drinking, or drug use. He had no sick contacts or recent travel. He explained that episodes similar to this had occurred in the last month prior to presentation but were never this severe and had previously resolved without intervention after several minutes.

Regarding medication use, he took daily vitamin D, vitamin B6, and vitamin B12 and had no change in the use of these vitamins for several years. He also noted that he recently began to use a supplement which he purchased over the internet called *Epimedium*, also known as horny goat weed, for the purpose of improving his mood and sense of energy and reducing his sense of anxiety. He noted that he first noted the onset of muscle cramps and spasms one month prior to ED presentation, coincident with the initiation of *Epimedium*. The episodes had become more prolonged and severe over the previous month. 

On physical exam, the patient had involuntary muscle contractions throughout his body. He was diaphoretic, writhing, and restless. He persistently asked staff to stretch out his extremities to help relieve his pain. His vital signs showed a heart rate of 110 beats per minute but were otherwise within normal limits. His physical examination was otherwise within normal limits. After receiving 1 mg of lorazepam, 15 mg of ketorolac, and a two-liter bolus of normal saline solution, his symptoms were relieved. His heart rate normalized.

An electrocardiogram (ECG) was within normal limits. His chest X-ray was unremarkable. Basic laboratory testing was obtained. His creatinine was 1.23 mg/dL in the ED, and his creatinine kinase (CK) was 1,210 U/L. A basic metabolic panel showed blood glucose of 186 mg/d. His magnesium level, complete blood count, and urinalysis were within normal limits. His lactate was elevated at 3.9 mmol/L. A urine drug screen was negative (Table 1).

**Table 1:** Emergency department laboratory results BUN: blood urea nitrogen; CK: creatine kinase; PT: prothrombin time; PTT: partial thromboplastin time; INR: international normalized ratio; NA: not applicable; K/uL: 1000 per microliter; g/dL: grams per deciliter; mEq/L: milliequivalents per liter; mg/dL: milligrams per deciliter; mcg/ml: micrograms per milliliter; cells/HPF: cells per high-power field; mmol/L: millimoles per liter; U/L: units per liter; sec: seconds

Laboratory results in the emergency department	Result	Normal range	Units
White blood cell count	10.8	4-11	K/uL
Hemoglobin	13	10.6-15.6	g/dL
Platelet count	160	150-400	K/uL
Sodium	137	135-154	mEq/L
Potassium	3.7	3.5-5	mEq/L
BUN	16	5-20	mg/dL
Creatinine	1.23	0.6-1.2	mg/dL
Glucose	186	70-100	mg/dL
Calcium	9	8.5-10.5	mg/dL
Chloride	100	95-105	mEq/L
Bicarbonate	28	23-29	mEq/L
Lactate	3.9	0.5-2.2	mmol/L
Magnesium	1.8	1.7-2.2	mg/dL
Phosphate	2.8	2.5-4.5	mg/dL
CK	1201	20-30	U/L
PT	11	11-13	sec
PTT	33	25-35	sec
INR	1	0.8-1.1	NA
Urine color	Yellow	Yellow	NA
Urine clarity	Clear	Clear	NA
Urine-specific gravity	1.012	1.005-1.030	NA
Urine pH	7	5-7.5	NA
Urine glucose	Negative	Negative	NA
Urine protein	Negative	Negative	NA
Urine bilirubin	Negative	Negative	NA
Urine urobilinogen	Negative	Negative	NA
Urine ketones	Negative	Negative	NA
Urine blood	Negative	Negative	NA
Urine white cells	Negative	0-5/HPF	cells/HPF
Urine red cells	Negative	0-5/HPF	cells/HPF
Urine nitrite	Negative	Negative	NA
Urine leukocyte esterase	Negative	Negative	NA
Urine drug screen	Negative	Negative	NA
Blood culture	No growth	No growth	NA

The patient was admitted to the hospital for further management of his muscle cramps and the management of his CK. His creatinine was 1.44 mg/dL the following morning, and his blood sugar normalized. His creatinine gradually trended to his baseline of 1.02 mg/dL. His lactate normalized. His CK trended up to a maximum of 3,271 U/L on hospital day 5 and trended down to normal by day 7. His blood cultures were negative. During admission, an autoimmune screening workup was negative, including anti-SS-A, anti-SS-B, ANA screen, and anti-dsDNA antibody. Computed tomography (CT) scan of the chest, abdomen, and pelvis was unremarkable. While an inpatient, he received magnetic resonance imaging (MRI) of the brain and cervical spine, which showed no abnormalities. Muscle cramp symptoms resolved completely by hospital day 6. On hospital day 7, he was discharged with methocarbamol for any recurrent spasms. He was given instructions to stop taking horny goat weed supplements. He was advised to follow up with primary care and neurology if his muscle spasms recurred.

## Discussion

*Epimedium* is sold in health stores and on the internet, in a variety of forms, using various species and sometimes in combination with known vitamins and other botanicals [2].

Clinical trials have been conducted on the use of *Epimedium *species for the treatment of a wide variety of conditions, including hypertension and coronary artery disease, and as an aphrodisiac. However, no randomized clinical trial-level data appear to exist in the literature [2]. One of the most commonly studied components of *Epimedium *species is icariin, which is a flavonol glycoside compound. Icariin has been suggested to increase nitrous oxide synthesis in the penis and to inhibit phosphodiesterase type 5 (PDE5) in the cavernosal smooth muscle [2]. It has also been studied in reference to possible anti-inflammatory, anti-osteoporotic, and neuroprotective activity [2].

Several cases have been published concerning toxic reactions to *Epimedium*. One case describes new-onset tachycardia and hypomania after two weeks of *Epimedium* use [3]. Abdominal pain and nausea have been reported as well [1]. A case of increased opiate cravings in a patient on buprenorphine has been reported. The purported mechanism was felt to relate to the induction of CYP3A4 metabolism. Buprenorphine is also metabolized by CYP34A [4].

It has been recommended that all patients be asked about the use of herbal supplements [4]. The absence of randomized clinical trial data on the safety and effectiveness of *Epimedium *species supplements has been noted by several authors, including concerns for drug-drug interactions [5,6].

In reference to muscle spasms, it has been noted by Jin et al. that icariin has a number of effects on the nervous system, but without specific reference to muscle spasms [7]. Several articles propose a number of effects of icariin on muscle cells, but without specific reference to muscle spasms [8,9]. To our knowledge, the patient presented here is the first medical case report of severe muscle spasms with associated elevations of CK and creatinine associated with *Epimedium* use.

In reference to other causes of an elevated CK, the patient denied recent strenuous exercise or trauma. There was no history of seizures, prolonged immobilization, or pressure-related injuries. He was not taking statins or other medications known to cause myopathy, such as fibrates or antipsychotics. He reported no symptoms suggestive of inflammatory myopathies or a personal or family history of muscular dystrophy. He denied illicit drug use, excessive alcohol consumption, or recent viral illness.

Mechanistic pathways and clinical implications of *Epimedium* and icariin

Potential Cardiovascular Protection via PI3K/Akt/eNOS and MEK/ERK Pathways

Icariin, the principal flavonoid in *Epimedium*, promotes angiogenesis by activating the PI3K/Akt/eNOS and MEK/ERK signaling pathways in human endothelial cells. This activation leads to increased nitric oxide (NO) production, enhancing endothelial function. This positions it as a possible target for cardiovascular protection research [10].

Anti-inflammatory Effects Through Glucocorticoid Receptor Modulation

Icariin exerts anti-inflammatory effects by upregulating glucocorticoid receptor alpha (GRα) expression and promoting its nuclear translocation. This modulation leads to the suppression of pro-inflammatory transcription factors, resulting in the decreased production of inflammatory cytokines like IL-6 and TNF-α. Icariin's ability to modulate GRα and suppress pro-inflammatory transcription factors positions it as a candidate for research in the management of chronic inflammatory conditions [11].

Neuroprotective Actions in Alzheimer's Disease Models

In Alzheimer's disease models, icariin ameliorates cognitive deficits by modulating multiple pathways, including BACE-1, NO/cyclic guanosine monophosphate (cGMP), Wnt/Ca²⁺, and PI3K/Akt signaling. It also inhibits neuronal apoptosis through the suppression of endoplasmic reticulum stress and attenuates neuroinflammation by inactivating microglial activity via the upregulation of PPARγ and inhibition of NF-κB and MAPK pathways. The neuroprotective properties of icariin, including inhibition of neuronal apoptosis and attenuation of neuroinflammation, indicate potential for research in such diseases as Alzheimer's [12].

Antidiabetic Properties

Icariin metabolites, such as icaritin and icariside II, exhibit potent inhibitory activities against protein tyrosine phosphatase 1B (PTP1B) and α-glucosidase. These enzymes are involved in insulin signaling and carbohydrate digestion, respectively. The inhibition of these enzymes suggests potential antidiabetic effects of icariin and its derivatives. Through the inhibition of PTP1B and α-glucosidase, icariin and its metabolites have the potential to improve insulin sensitivity and glycemic control, indicating the potential for further research [13].

PDE5 Inhibition and Erectile Function Enhancement

Icariin and its derivatives inhibit PDE5, leading to increased levels of cGMP and enhanced blood flow, which may improve erectile function [14].

Possible Anticancer Activity Through Apoptosis Induction

Icariin induces apoptosis in various cancer cell lines, including lung adenocarcinoma and tamoxifen-resistant breast cancer cells, by activating the mitochondrial apoptotic pathway and suppressing autophagy. These effects are mediated through the modulation of the PI3K/Akt pathway and the regulation of apoptosis-related proteins. The pro-apoptotic and autophagy-suppressing effects of icariin suggest potential for research in cancer treatment [15,16].

Comparative analysis of *Epimedium *with selected similar herbal supplements (ginseng, *Tribulus terrestris*, and *Ginkgo biloba*)

*Epimedium*, ginseng, *Tribulus terrestris*, and *Ginkgo biloba* each possess unique pharmacological profiles. While there are overlapping mechanisms, such as PI3K/Akt pathway activation, distinctions exist in their specific actions and therapeutic applications.

Ginsenosides, the active components of ginseng, also activate the PI3K/Akt pathway, contributing to cardiovascular protection and neuroprotection. However, ginsenosides primarily exert their effects through the modulation of NO production and antioxidant properties. While both icariin and ginsenosides share similar pathways, icariin's PDE5 inhibitory activity distinguishes it in the context of erectile dysfunction research [17].

*Tribulus terrestris* is traditionally used to enhance libido and sexual function. Its active compounds, such as protodioscin, are believed to influence androgen levels. Unlike icariin, which directly inhibits PDE5, the mechanisms of *Tribulus terrestris* are less well-defined and lack the enzyme-specific actions observed with icariin [18].

*Ginkgo biloba* exhibits possible neuroprotective effects through antioxidant activity and the modulation of neurotransmitter systems. While it shares some pathways with icariin, such as PI3K/Akt activation, ginkgo's primary mechanisms involve free radical scavenging and the inhibition of platelet-activating factor, differing from icariin's multifaceted actions [19].

Thus, the multifaceted pharmacological actions of icariin suggest potential therapeutic applications in various clinical settings. However, while preclinical studies are promising, clinical trials are necessary to validate these effects in humans and determine appropriate dosing, safety, and efficacy.

## Conclusions

This case describes a 33-year-old man who developed severe muscle spasms with elevated CK and creatinine following the use of *Epimedium*, an over-the-counter supplement. Symptoms and laboratory abnormalities resolved with supportive care and discontinuation of the supplement. To our knowledge, this is the first reported case linking *Epimedium* to such findings. The case highlights the importance of thorough medication histories, including non-prescription and herbal supplements. 
